# Depression-associated gut microbes, metabolites and clinical trials

**DOI:** 10.3389/fmicb.2024.1292004

**Published:** 2024-01-31

**Authors:** Meiling Wang, Zhaoqi Song, Shirong Lai, Furong Tang, Lijun Dou, Fenglong Yang

**Affiliations:** ^1^Department of Bioinformatics, Fujian Key Laboratory of Medical Bioinformatics, School of Medical Technology and Engineering, Fujian Medical University, Fuzhou, China; ^2^Department of Basic Medical Sciences, School of Medicine, Tsinghua University, Beijing, China; ^3^Genomic Medicine Institute, Lerner Research Institute, Cleveland, OH, United States; ^4^Key Laboratory of Ministry of Education for Gastrointestinal Cancer, School of Basic Medical Sciences, Fujian Medical University, Fuzhou, China

**Keywords:** depression, gut microbiota, metabolites, pathogenesis, clinical trials

## Abstract

Depression is one of the most prevalent mental disorders today. Over the past decade, there has been considerable attention given to the field of gut microbiota associated with depression. A substantial body of research indicates a bidirectional communication pathway between gut microbiota and the brain. In this review, we extensively detail the correlation between gut microbiota, including *Lactobacillus acidophilus* and *Bifidobacterium longum*, and metabolites such as short-chain fatty acids (SCFAs) and 5-hydroxytryptamine (5-HT) concerning depression. Furthermore, we delve into the potential health benefits of microbiome-targeted therapies, encompassing probiotics, prebiotics, and synbiotics, in alleviating depression. Lastly, we underscore the importance of employing a constraint-based modeling framework in the era of systems medicine to contextualize metabolomic measurements and integrate multi-omics data. This approach can offer valuable insights into the complex metabolic host-microbiota interactions, enabling personalized recommendations for potential biomarkers, novel drugs, and treatments for depression.

## Highlights

- This paper reviews reported microbiota-based animal studies related to depression and microbiome-based clinical trials in depression. We also summarized the different combinations of therapies and the resulting efficacy, and explored possible reasons for the poor efficacy.
- We briefly describe certain gut microbial metabolites that influence the onset and progression of depression via MGB axis, including 5-HT, SCFAs, GABA, GP, choline, lactate, BAs and vitamin (folate).
- This paper proposes metabolic models as a means of testing new hypotheses and exploring new microbial-based therapies for depression.

## Introduction

Depression is the most common mental disorder today, characterized by persistent and prolonged feelings of sadness as its main clinical feature. It has a high prevalence, high recurrence rate, and a tendency towards suicide (Belmaker and Agam, 2008), making it one of the most significant types of psychological disorders in modern society. Epidemiological studies from various countries indicate a depression prevalence rate of approximately 7% in the general population (For Anxiety and Depression, R. G. for Treatment, 2017), and up to 10% of individuals with depression may attempt suicide (Bachmann, 2018). Major depressive disorder (MDD) has a devastating impact on global public health, not only causing social and economic burdens but also being a leading cause of severe disability (World Health Organization, 2008). The World Health Organization has identified depression as one of the top public health priorities, estimating that by 2030, it will be the leading cause of death and disability in Western countries (For Anxiety and Depression, R. G. for Treatment, 2017).

Over the past decade, there has been a growing interest in the relationship between gut microbiota and mental disorders (Sanada et al., 2020). The gut microbiota ecosystem contains approximately 1 kg of bacteria, encompassing all microorganisms, their genes, encoded proteins, and metabolites (Eckburg et al., 2005; Korecka and Arulampalam, 2012). Additionally, the gut houses a large number of neurons, second only to the brain, earning the nickname “the second brain” (Ridaura and Belkaid, 2015). Extensive research has established the microbiota-gut-brain (MGB) axis (Bercik, 2011; Camilleri et al., 2012). Along this axis, the microbial community in the gut influences brain function through three bidirectional signaling pathways (Macpherson and Harris, 2004; Bäckhed et al., 2005; Heijtz et al., 2011; Cryan and Dinan, 2012; Breit et al., 2018). Alterations in these signaling pathways may contribute to mental health problems. Therefore, investigating the MGB axis provides a novel approach to exploring the pathogenesis of depression and developing appropriate therapeutic strategies.

In this review, we will summarize the recent advances in gut microbiota research related to depression, focusing on the relationship and role of the MGB axis in depression. We aim to explore the value and potential of the MGB axis in the diagnosis of depression.

## Gut microbes in depression

There are several pathophysiological hypotheses explaining depression, including the monoamine, brain-derived neurotrophic factor (BDNF), and cytokine hypotheses (Boku et al., 2018). However, these hypotheses have their limitations. Increasing evidence suggests that the gut microbiota may play a role in depression. In a study by McGuinness et al. (2022), it was found that the α-diversity did not significantly differ between the majority of MDD cases and the control group, with only a few reports indicating higher or lower α-diversity between the two groups. However, when statistically tested using β-diversity analysis, 87% of MDD cases showed differences in gut microbiota composition compared to the control group. The study identified 21 bacterial genera with differential abundance at the genus level. Higher abundances of *Alistipes*, *Parabacteroides*, *Streptococcus*, *Veillonella*, *Enterococcus*, *Flavonifractor*, *Eggerthella*, *Escherichia*, and lower abundances of *Coprococcus*, *Prevotella*, *Faecalibacterium*, and *Ruminococcus* were observed in MDD cases. Among them, higher proportions of *lactobacilli* and lactic acid-producing bacteria, which are generally considered beneficial to the host, were found. These bacteria promote gut microbiota balance (Shi et al., 2017), maintain a normal microbial environment, and have immunomodulatory effects (Pessione, 2012; George et al., 2018). However, in certain circumstances, the production and utilization of lactate can also have detrimental effects on host health. Lactate can accumulate in the gut and cross the blood–brain barrier (Proia et al., 2016), potentially leading to acidosis, arrhythmias, and neurotoxicity (Duncan et al., 2004; Pham et al., 2017). Many psychiatric disorders are also associated with mitochondrial energy dysfunction (Regenold et al., 2009), indicated by increased lactate and decreased pH in the brain. Elevated levels of lactate have been observed in the brains of MDD patients (Ernst et al., 2017), suggesting that an increase in the abundance of lactate-producing bacteria and subsequent lactate accumulation may contribute to the pathophysiology of depression. Additionally, there is evidence suggesting an association between *Clostridium difficile* and the onset of depression. In a study conducted by Fondden et al. and published in the journal “Nutrients” in 2020, a significant finding indicated that an elevated presence of *C. difficile* is associated with an increased risk of depression. The study compared individuals with depression to healthy individuals and discovered a 36% higher abundance of *C. difficile* in those with depression. However, it was also observed that fecal microbiota transplantation proved to be an effective method in reducing the levels of *C. difficile*, thereby inhibiting the occurrence of depression (van Nood et al., 2013; Austin et al., 2014; Cammarota et al., 2015; Li et al., 2016; Hocquart et al., 2018; Fond et al., 2020). The recent study published in ‘Nature Communications’ provide some of the most compelling evidence to date regarding the relationship between depression and gut microbiota (Radjabzadeh et al., 2022). One study, known as HELIUS, specifically examined health disparities among individuals of different racial backgrounds living in the same urban environment, with the primary aim of investigating the general association between the microbiome and depression. Interestingly, one of the studies within this research did indeed uncover variations in depression risk among different racial groups, but these differences could be explained by individual variations in the composition of one’s microbiome. Overall, the study found a consistent association between overall microbial diversity and depression, transcending racial boundaries. The second study delved more specifically into the types of gut bacteria that may be linked to depression. In a meticulous analysis of fecal samples from approximately 1,000 participants in an ongoing population health study in Rotterdam, 13 microbial species were directly associated with symptoms of depression. The most significant new discovery in this research was the connection between *Sellimonas* and depression symptoms. Bacterial species belonging to the *Sellimonas* genus are involved in various inflammatory diseases, potentially linking them to inflammation in individuals with depression. These findings suggest that a causal relationship between the microbiome and depression is entirely plausible, and it is reasonable to consider that depression may lead to other physiological changes, subsequently altering the microbiome (Radjabzadeh et al., 2022).

Multiple studies have demonstrated the involvement of the gut microbiota in the occurrence and development of psychiatric disorders, including depression (Desbonnet et al., 2010; Zheng et al., 2016; Rincel et al., 2019; Pearson-Leary et al., 2020). Transplanting microbiota from depressed patients into normal animals has been found to induce depression-like behaviors (Bravo et al., 2011; Savignac et al., 2014; Liang et al., 2015; Campos et al., 2016; Gacias et al., 2016; Kelly et al., 2016; Zheng et al., 2016). Rats receiving fecal microbiota transplantation from depressed patients displayed anhedonia-like behavior in a sucrose preference test (Kelly et al., 2016). Germ-free mice colonized with microbiota from depressed patients showed increased immobility time in tail suspension and forced swim tests, along with an increased abundance of Actinobacteria, compared to mice colonized with microbiota from healthy individuals (Zheng et al., 2016). These findings, supported by a substantial body of evidence, suggest that alterations in the gut microbiota can contribute to the onset of depression.

**Figure 1:**
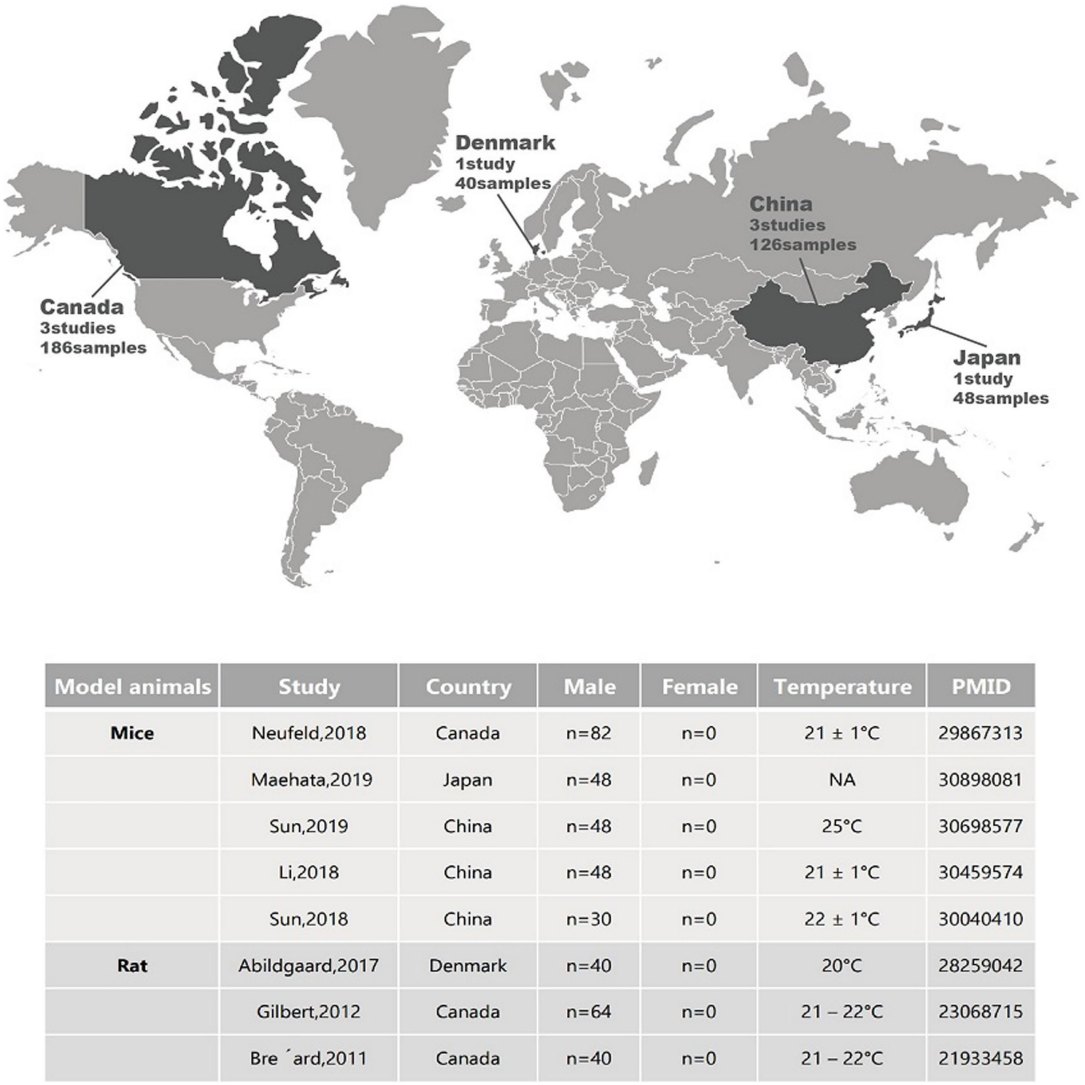
Geographical locations and sample metadata of different animal studies related to depression.

While changes in the gut microbiota can contribute to the onset of depression, the gut microbiota also has the potential to improve depressive symptoms. Animal studies (Figure 1) have shown that both antibiotics (Gacias et al., 2016; Guida et al., 2018) and probiotics (Bravo et al., 2011) can significantly alter depression-like behaviors in rats and mice, demonstrating the beneficial effects of gut microbiota as probiotics in alleviating depressive symptoms (Table 1). For instance, in rat studies, a combination therapy of eight probiotic strains (*B. bifidum* W23*, B. lactis* W52, *L. acidophilus* W37*, L. brevis* W63, *L. casei* W56, *L. salivarius* W24, *Lactococcus lactis* W19, *Lactococcus lactis* W58) significantly reduced diet-independent depression-like behaviors (Abildgaard et al., 2017). In mouse studies, supplementation of *L. helveticus* MCC1848 significantly increased interaction time in the social interaction test and sucrose preference ratio in the sucrose preference test (Maehata et al., 2019). Oral administration of *L. kefiranofaciens* ZW3 improved depression-like behaviors and independent exploration ability, regulating biochemical disorders in the hypothalamic–pituitary–adrenal axis, immune system, and tryptophan metabolism (Sun et al., 2019). *Clostridioides butyricum* demonstrated significant effects by increasing 5-HT and glucagon-like peptide-1 (GLP-1), upregulating BDNF expression, and promoting GLP-1 secretion and GLP-1 receptor expression (Sun et al., 2018) and GLP-1 has been reported to possess the potential to alleviate depression by regulating neuroinflammation, neurotransmitters, neurogenesis, and synaptic function (Kim et al., 2020). Furthermore, treatment with a multi-strain probiotics approach (*L. helveticus* R0052, *L. plantarum* R1012, and *B. longum* R0175) attenuated anxiety and depression-like behaviors induced by chronic mild stress, significantly increased *Lactobacillus* abundance, and reversed immune changes in the hippocampus induced by chronic mild stress (Li et al., 2018). Mice subjected to a series of stress stimuli exhibited depression-like behaviors and dysbiosis of the microbiota, but this condition could be reversed by probiotic administration, supplementation with gut bacteria, or antibiotic treatment. Mice subjected to chronic social defeat stress and treated with prebiotics or *Bifidobacterium* orally showed a reduction in depression-like behaviors in tests such as tail suspension and forced swim (Burokas et al., 2017; Yang et al., 2017). Similarly, mice and rats subjected to chronic restraint stress and treated with minocycline or oral *L. helveticus* NS8, respectively, reversed the increased depression-like behaviors and altered gut microbiota induced by chronic restraint stress (Liang et al., 2015; Wong et al., 2016). Studies involving mice and rats in models of unpredictable chronic mild stress (Marin et al., 2017), learned helplessness (Mika et al., 2017; Takajo et al., 2019), and maternal separation (Zheng et al., 2016) observed a reduction in depression-like behaviors following treatment with probiotics and supplementation with gut bacteria, while untreated mice and rats showed changes in fecal metabolomic profiles associated with depression-like behaviors (O’Mahony et al., 2009; Jianguo et al., 2019; Zhang et al., 2019). These findings from animal models of depression collectively emphasize the significant role of the gut microbiota and underscore the importance of animal models in microbiota research.

**Table 1:** Antidepressant effects of probiotics in animal studies.

Model animals	Probiotics	Administration form	Finding	PMID
Mice	*Lactobacillus (L.) rhamnosus* JB-1™	Oral administration via drinking water	Adult male BALB/c mice responded with greater antidepressive-like behavior to probiotic while SW mice did not.	29867313
	*L. helveticus* strain MCC1848	Oral intake of heat-killed probiotics	MCC1848 supplementation significantly enhanced interaction time in social interaction test and sucrose preference ratio in the sucrose preference test.	30898081
	*L. kefiranofaciens* ZW3	Oral administration	ZW3 improved depression-like behavior and independent exploration ability, regulated biochemical disorders in the hypothalamic–pituitary–adrenal axis, immune system, and tryptophan metabolism. Probiotic strain stayed in intestine 7 days after intervention ceased.	30698577
	*L. helveticus* R0052*, L. plantarum* R1012*, and Bifidobacterium (B.) longum* R0175	Oral administration	Probiotics attenuated CMS-induced anxiety-and depressive-like behaviors, significantly increased Lactobacillus abundance, and reversed the CMS-induced immune changes in the hippocampus.	30459574
	*Clostridioides butyricum*	Gavage administration	Clostridioides butyricum exhibited prominent effects, increasing 5-HT and GLP-1 and upregulating BDNF expression, and secretion of GLP-1 and upregulated GLP-1R expression.	30040410
Rat	*B. bifidum* W23, *B. lactis* W52, *L. acidophilus* W37, *L. brevis* W63, *L. casei* W56, *L. salivarius* W24, *Lactococcus lactis* W19, *Lactococcus lactis* W58	Oral administration via drinking water	Multispecies probiotics treatment markedly reduced depressive-like behavior independently of diet.	28259042
	*L. helveticus* R0052 and *B. longum* R0175	Gavage administration	Probiotics attenuated post-myocardial infarction depression as well as n-3 fatty acids did.	23068715
	*L. helveticus* R0052 and *B. longum* R0175	Oral administration via drinking water	Probiotics interferes with the development of post-MI depressive behavior and restores intestinal barrier integrity in MI rats.	21933458

Details of the samples used in theses studies can be found in Figure 1.

## Metabolites in depression

**Figure 2:**
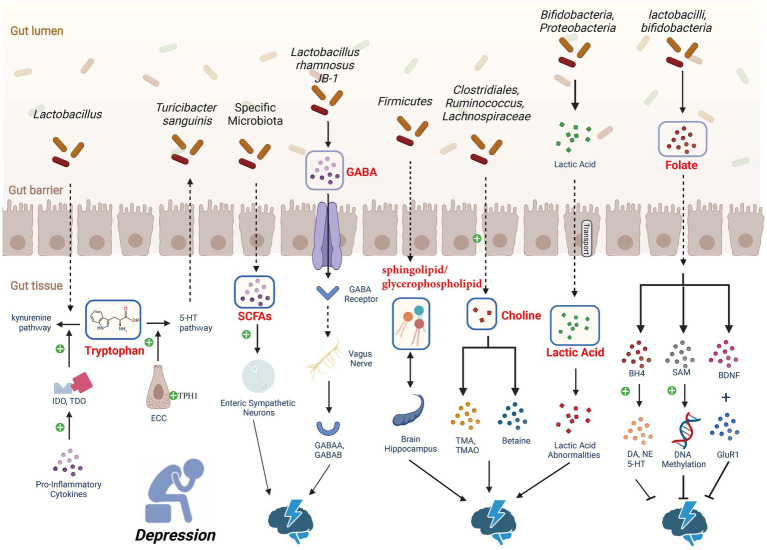
Depression-associated metabolic pathways. Gut microbiota regulates the levels of 5-HT to influence depression. Through dietary or drug interventions, it upregulates the expression of tryptophan hydroxylase 1 (TPH1) in enterochromaffin cells (ECCs), enhancing the production of 5-HT. SCFAs have been shown to affect the production of 5-HT in the gut. An increase in dietary tryptophan induces the synthesis of SCFAs by gut microbiota, leading to an increase in 5-HT production and release in ECCs. Gut microbiota has the ability to increase γ-aminobutyric acid (GABA) levels in the central nervous system (CNS) of mice. GABA crosses the intestinal barrier and is sensed by the vagus nerve, transmitting signals to paraventricular nucleus (PVN) neurons, thereby initiating hypothalamic–pituitary–adrenal (HPA) axis activity and producing an antidepressant effect. In the human body, choline positively influences emotions through promoting SAM-dependent DNA methylation. However, oral choline increases the concentration of acetylcholine in the brain, promoting depression-like behavior. Choline deficiency or excess may both affect depression, highlighting the complexity of the relationship between choline metabolism and depressive behavior. Lactic acid can pass through the blood–brain barrier, and studies in rodents and humans have found a connection between depression and lactic acid abnormalities. About one-third of depression patients exhibit folate deficiency. Folate supplementation has demonstrated its antidepressant effects, but clinical trials have not yet provided strong evidence to support folate as an advantageous adjunctive strategy for depression. The signaling pathway of bile acid metabolism within the gut–liver axis (not depicted in the figure), subject to structural modifications by the gut microbiota, can elicit either severe depression or manifest antidepressant effects based on the specific receptors involved. Created with BioRender.com.

Certain gut microbial metabolites have been shown to influence the occurrence and progression of depression through the MGB axis (Figure 2).

### Tryptophan metabolism

One important factor in depression is tryptophan, and a study focusing on the impact of dietary tryptophan on mood disorders emphasizes that a diet rich in tryptophan helps reduce depressive symptoms and improve an individual’s emotional state. Conversely, a low tryptophan diet can lead to irritability and anxiety (Lindseth et al., 2015). Tryptophan can be metabolized in two crucial pathways of depression: the 5-HT and kynurenine pathways (Comai et al., 2020).

As a key modulator of the gut-brain axis, the neurotransmitter 5-HT plays a crucial role in the communication between the gut and the brain in the signaling of the MGB axis. Recently, an increasing number of studies have indicated the significant role of 5-HT, including its precursor 5-hydroxytryptophan (5-HTP), in the development of depression. Tryptophan hydroxylase (TPH) enzyme plays an important role in various psychiatric disorders, including depression. Research suggests that stress suppresses the expression of this enzyme, thereby reducing the levels of 5-HT (Chen et al., 2017b). Peripheral cells involved in the production of 5-HT exhibit TPH1 dysfunction, leading to insufficient levels of 5-HT in the brain, which in turn triggers a homeostatic response of TPH2, an enzyme that is overexpressed in individuals with suicidal tendencies, in response to low 5-HT levels (Bach-Mizrachi et al., 2006; Fukuda, 2014). This suggests that low 5-HT may contribute to the development of depression. The gut microbiota, in turn, influences depression by regulating the levels of 5-HT. Antibiotic-treated or germ-free mice exhibit reduced synthesis of 5-HT, which can be reversed by the colonization of spore-forming bacteria (Yano et al., 2015). Specific spore-forming bacteria from humans and mice increase the levels of 5-HT in the colon and serum of germ-free mice through the production of SCFAs, thereby upregulating the expression of TPH1 in ECCs and enhancing 5-HT production (Reigstad et al., 2015; Yano et al., 2015). This also improves gut motility disorders associated with germ-free conditions (Furusawa et al., 2013). The communication between ECCs-released 5-HT and the gut microbiota *Turicibacter sanguinis*, which possesses a 5-HT uptake mechanism, is involved in its colonization and host physiology (Fung et al., 2019). The most common antidepressant medications are based on blocking the reuptake of 5-HT, thereby increasing its levels in the synaptic cleft and promoting antidepressant responses (Cowen and Browning, 2015; Vahid-Ansari and Albert, 2021). This explains the bidirectional effects observed between certain psychotropic drugs, including selective serotonin reuptake inhibitors, and the gut microbiota (Cussotto et al., 2019), suggesting the facilitatory role of high concentrations of 5-HT in antidepressant effects. Furthermore, depression patients show inadequate transport of 5-HTP to the brain (Ryan, 1992; Maffei, 2020), and another study also suggests that the combination of 5-HTP with niacinamide is more effective in combating depression compared to niacinamide alone (López-Ibor Aliño et al., 1976; Maffei, 2020), highlighting the impact of 5-HTP on depression.

Another pathway of tryptophan metabolism is the kynurenine pathway. Excessive pro-inflammatory cytokines produced in depression over activate the enzymes indoleamine 2,3-dioxygnease (IDO) and tryptophan 2,3-dioxygenase (TDO), promoting the kynurenine pathway and consequently reducing the activation of the 5-HT pathway and decreasing the production of 5-HT (Miura et al., 2008). It is worth mentioning that IDO and TDO inhibitors, by inhibiting the activation of IDO and TDO enzymes, can serve as potential drugs for the treatment of depression (Qin et al., 2018). Studies on germ-free mice have shown that the availability of tryptophan increases due to reduced activation of the peripheral kynurenine pathway (Clarke et al., 2013). Moreover, in a rodent model of chronic unpredictable stress, the decrease in stress-induced *lactobacillus* abundance weakens the inhibition of IDO 1 mediated by hydrogen peroxide. This inhibition leads to an increase in the conversion of tryptophan to kynurenine, resulting in behavioral changes resembling depression in mice exposed to chronic stress (Marin et al., 2017). In contrast to 5-HT, kynurenine can cross the blood–brain barrier and negatively impact brain health through the induction of neuroinflammation and neurodegenerative changes (Kennedy et al., 2017). Therefore, the study of 5-HT and kynurenine metabolism in tryptophan metabolism holds significant importance in depression research, and the balance between the two is closely linked to the physiopathology of depression.

### SCFAs

In the gut, short-chain fatty acids (SCFAs) serve as common microbial metabolites and are closely associated with depression. Reports indicate that SCFAs are depleted in patients with MDD (Zheng et al., 2016; Skonieczna-Żydecka et al., 2018). However, administration of SCFAs, especially butyrate, has been shown to improve depression-related gut permeability and HPA axis reactivity, resulting in antidepressant effects (Van De Wouw et al., 2018; Caspani et al., 2019). Research related to depression suggests that the expression of BDNF, can be altered by exogenous SCFAs. Long-term administration of exogenous sodium butyrate in mice has significantly reduced depressive-like behavior, indicating that SCFAs may influence the occurrence and development of depression through their effects on the brain (Schroeder et al., 2007).

SCFAs impact the activity of the enteric nervous system (ENS) and regulate intestinal motility in rodents through free fatty acid receptors present on epithelial cells, enteroendocrine cells (EECs), ECCs, immune cells, and endogenous and exogenous neurons (Muller et al., 2020). These pathways modulate local neuronal cells in the metabolism and/or ENS and the afferent pathway of the vagus nerve, which directly signals to the brain (Morais et al., 2021). A study showed that germ-free mice, lacking gut microbiota, exhibited increased activation of extrinsic neurons connecting the brainstem sensory and enteric sympathetic neurons. However, the activation of these neurons was inhibited by the administration of gut microbiota that produces SCFAs. These findings suggest that gut microbiota can regulate the gut-brain axis neuronal pathway through SCFAs. Additionally, SCFAs have been shown to affect the production of intestinal 5-HT (Bonaz et al., 2018). In humans and mice, increased dietary tryptophan induces the synthesis of SCFAs by gut microbiota, leading to increased production and release of 5-HT in ECCs and enhanced gastrointestinal motility (Reigstad et al., 2015; Yano et al., 2015; Agus et al., 2018; Yu et al., 2019).

However, SCFAs have a relatively short half-life (25 min to 3 h), and further research is needed to determine the extent of the influence of physiologically relevant concentrations of SCFAs on the brain (Margolis et al., 2021).

### GABA

γ-aminobutyric acid (GABA) is a naturally occurring amino acid found widely in vertebrates, plants, and microorganisms. It is an important inhibitory neurotransmitter in the central nervous system (Rashmi et al., 2018). In recent probiotic research, it has been reported that GABA can be produced by gut microbiota, following a synthesis pathway similar to that in the central nervous system (Janik et al., 2016), and has been shown to alleviate depressive-like behavior in mice (Bravo et al., 2011).

Studies have indicated that certain gut microbiota have the ability to increase GABA levels in the central nervous system of mice, thereby modulating depressive-like behavior. One such microorganism is *L. rhamnosus* JB-1 (Yunes et al., 2016; Strandwitz et al., 2018), which produces GABA that can cross the intestinal barrier via the proton-coupled amino acid transporter hPAT1 (Nielsen et al., 2012; Bonaz et al., 2017; Yong et al., 2020) and is sensed by the vagus nerve (Bonaz et al., 2017). The vagus nerve activates the GABA signaling pathway to regulate the expression of GABAA and GABAB receptors (Yunes et al., 2016; Strandwitz et al., 2018), allowing the GABA produced by the microbiota to interact with the widely expressed GABA receptors and transporters on the afferent neurons of the vagus nerve (Nielsen et al., 2012; Yong et al., 2020). Additionally, the vagus nerve initiates neural activation in the nucleus tractus solitarius (NTS) of the central nervous system. Sensory gut information transmitted to the NTS is then integrated into its extensive projections, such as the PVN of the hypothalamus, where PVN neurons are responsible for initiating HPA axis activity and producing antidepressant effects (Bravo et al., 2011; Janik et al., 2016).

### Glycerophospholipids

Some evidence suggests that the host’s lipid metabolism is influenced by the gut microbiota (Blaszczak et al., 2019). Lipids play a crucial role in neuronal function, and the lipid composition of the brain may impact perception and emotional behavior, potentially leading to depression and anxiety (Adibhatla and Hatcher, 2008; Yadav and Tiwari, 2014; Kornhuber et al., 2015). Glycerophospholipids (GP) are major structural lipid components of eukaryotic cell membranes and are involved in numerous cellular processes. Disruption of the gut microbiota, as observed in germ-free mouse experiments, may induce depressive-like behavior by modulating host metabolism (Zheng et al., 2016). Further research has revealed that the gut microbiota primarily influences host GP metabolism (Tian et al., 2022). An experiment found that the hippocampus exhibited the highest degree of disruption in lipid metabolic pathways. The differentially metabolized compounds in the hippocampus were mainly enriched in GP metabolites, with a small proportion belonging to sphingolipid metabolism. Compared to the healthy control (HC) group, the depressive-like (DL) group showed upregulation of most hippocampal metabolites involved in GP metabolism. Furthermore, two metabolites involved in sphingolipid metabolism (dihydroceramide and ceramide-1-phosphate) were significantly decreased in the DL group compared to the HC group. In a chronic unpredictable mild stress rat model of depression, a decrease in lipid metabolism-related enzymes associated with fatty acid synthesis and metabolism, as well as GP metabolism, was observed (Oliveira et al., 2016). These findings indicate an imbalance in hippocampal sphingolipid and GP metabolism associated with depressive-like behavior (Zheng et al., 2021).

Consistent results from studies on humans and non-human primates indicate that dysbiosis of the bacterial phylum Firmicutes may be a hallmark of depression. Zheng et al. found that alterations in microbial and metabolic modules related to fatty acyl, sphingolipid, and GP metabolism were highly correlated with depressive-like behavior (Zheng et al., 2021). Within these microbial modules, several microbial genes involved in fatty acyl, sphingolipid, and GP metabolism were identified, suggesting that the gut microbiota and their regulated host metabolites may play a crucial role in the pathophysiology of depression. Interestingly, most of the unsaturated fatty acids used for the synthesis of brain neuronal membrane GP originate from the gastrointestinal tract rather than the central nervous system, indicating that GP metabolism via the gut-brain axis interferes with depression.

### Choline metabolites

Choline is a constituent of all biological membranes and a precursor of acetylcholine in cholinergic neurons. The acquisition of choline in the body occurs through food sources such as liver and eggs, primarily in the form of phosphatidylcholine (PC), or from endogenously synthesized PC through a continuous methylation process of phosphatidylethanolamine (PE). Choline itself is not a product of bacteria, but under the influence of the gut microbiota, choline can be metabolized into a series of compounds, including trimethylglycine (betaine) and trimethylamine (TMA). In the liver, TMA is converted to trimethylamine N-oxide (TMAO) by flavin monooxygenases (Dumas et al., 2006). Studies have found that the levels of TMA and TMAO in mouse plasma are positively correlated with *Clostridiales*, *Ruminococcus*, and *Lachnospiraceae* in the gut, while negatively correlated with the proportions of S24-7, an abundant family from *Bacteroidetes* (Wang et al., 2015). In the findings by Romano et al. (2017), choline and its metabolites were found to affect emotional behavior through DNA methylation. Choline regulates the production of the methyl donor S-adenosylmethionine (SAM) to promote DNA methylation. Bacterial consumption of choline reduces the availability of methyl donors, which is consistent with reports of decreased hippocampal DNA methylation and abnormal neurodevelopment in offspring due to maternal choline deficiency (Mellott et al., 2007). In the human body, betaine has a positive impact on emotions by promoting SAM-dependent DNA methylation (Di Pierro and Settembre, 2015). In a rat model of stress, supplementation of choline, betaine, and other methyl donors successfully reversed depressive-like behavior (Paternain et al., 2016).

However, oral administration of choline may promote depressive-like behavior. Choline can actively cross the blood–brain barrier (Sawada et al., 2010), and oral choline increases the concentration of acetylcholine in the brain (Babb et al., 2004), indicating that abnormal choline metabolism may promote depressive-like behavior by altering the choline concentration used for acetylcholine synthesis. Studies have shown that the concentration of the neurotransmitter acetylcholine is significantly higher in patients with MDD than in healthy subjects (Mineur and Picciotto, 2010). All of these findings indicate that choline and its metabolites have a significant impact on emotions through the gut microbiota. Choline deficiency can impair mental health, while excessive choline intake may lead to excessive synthesis of acetylcholine and result in depressive-like behavior. This also suggests the complexity of the relationship between choline metabolism and depressive behavior.

### Lactate

Lactic acid is produced through the fermentation of dietary fiber by mammalian hosts and specific bacteria such as lactic acid bacteria, *Bifidobacteria*, and *Proteobacteria* (Ríos-Covián et al., 2016). Although the concentration of lactate in the intestine is low, it can be absorbed into the bloodstream (Tahara et al., 2018) and can cross the blood–brain barrier (Knudsen et al., 1991). Studies in rodents and humans have indicated a connection between depression and lactate abnormalities (Carrard et al., 2018; Karnib et al., 2019), with increased urinary lactate levels observed in severe MDD patients (Chen et al., 2017a). In germ-free mice, elevated lactate concentrations have been observed in the hippocampus, while germ-free rats exhibit increased lactate concentrations in the frontal lobe (Swann et al., 2017).

Karnib et al. (2019) found that lactate salts have a protective and reversing effect on depression. Mice treated with lactate showed increased levels and activity of HDAC2/3 in the hippocampus, while mice not receiving lactate exhibited depressive-like behavior. The efficient exchange of lactate between the peripheral and central nervous systems (Knudsen et al., 1991) suggests the crucial role of the gut microbiota in mediating the antidepressant effects of lactate salts.

### Bile acids

Bile acids (BAs), synthesized from liver cholesterol, are pivotal end-products in cholesterol metabolism, constituting essential components of bile and primarily existing in the enterohepatic circulation system. In humans and rats, the main BAs are cholic acid (CA) and chenodeoxycholic acid (CDCA), subject to structural modifications by gut microbiota, leading to the formation of secondary and tertiary BAs (Russell, 2003). Higher levels of cytotoxic secondary BAs, derived from the primary bile acid CDCA by bacterial modifications, have been extensively reviewed in correlation with the severity of anxiety symptoms (Guzior and Quinn, 2021; Mahmoudian Dehkordi et al., 2022; Qu et al., 2022; Sun et al., 2022).

The signaling pathway of BAs is initiated by their binding to farnesoid X receptor (FXR) and Takeda G protein-coupled receptor 5 (Mertens et al., 2017). FXR is involved in bile acid synthesis, secretion, transport, and regulation of cAMP-response element binding protein (CREB) activity. By inhibiting the transcription factor CREB, BAs can suppress the transcription of BDNF, suggesting a potential influence of BAs on depression. Reports have indicated that the abnormal activity of BDNF in individuals with depression may be partially caused by changes in bile acid activity. In the chronic unpredictable mild stress (CUMS) rodent model of depression, an overexpression of FXR in the hippocampus has been observed. Conversely, overexpression of FXR in the rat hippocampus induces depression-like behavior, while the deletion of the FXR gene in juvenile rats inhibits the occurrence of depression-like behavior (Chen et al., 2018). An independent study has also confirmed the antidepressant effect of FXR gene deletion (Huang et al., 2015). Additionally, BAs may disrupt tight junction expression, leading to increased permeability of the intestinal and central epithelial cells, which can result in severe depression (Quinn et al., 2014). However, some BAs, such as ursodeoxycholic acid, have shown good antidepressant effects (Moore et al., 2000; Spedding, 2014), indicating that the impact of BAs on depression-like behavior may depend on specific receptors involved.

### Vitamin (folate)

Humans heavily rely on the gut microbiota, such as *lactobacilli* and *bifidobacteria*, to produce vitamins (Gu and Li, 2016), which play important roles in the human body. In the central nervous system, vitamins can influence neurotransmitter production (Kennedy, 2016), thereby impacting neuronal function.

Folate, a microbiota-derived vitamin, has been widely implicated in depression research (Brocardo et al., 2008, 2009; Molina-Hernández et al., 2011; McCoy et al., 2016; Paternain et al., 2016; Gao et al., 2017), with approximately one-third of individuals with depression showing folate deficiency (Miller, 2008). Administration of folate has demonstrated antidepressant effects in animal models of depression (Brocardo et al., 2008; Molina-Hernández et al., 2011; Gao et al., 2017), and some clinical studies have indicated its potential as an adjunctive therapy for depression in humans (Coppen et al., 1986; Coppen and Bailey, 2000). Folate can synthesize tetrahydrobiopterin, which acts as a cofactor for the conversion of phenylalanine and tryptophan into neurotransmitters dopamine, nzorepinephrine and 5-HT (Wolf et al., 1991), thereby enhancing serotonergic and noradrenergic activity in mice and exerting antidepressant effects. Furthermore, in addition to increasing central 5-HT, folate can induce increased expression of BDNF and glutamate receptor 1 in the hippocampus and associated cortex. The active metabolite of folate, 5-methyltetrahydrofolate, converts homocysteine into methionine, which is used as a methyl donor to produce S-adenosylmethionine (SAM). SAM has been shown to exert antidepressant effects through DNA methylation of phospholipids (Young and Ghadirian, 1989; Kagan et al., 1990; Bottiglieri et al., 1994). Although significant improvements in depression-like behavior have been observed in animal studies, clinical trials have not provided strong evidence supporting the advantage of folate as an adjunctive strategy for depression (Roberts et al., 2018).

The effects of the above metabolites on depression, relevant mechanisms, and reference information have been summarized in Table 2.

**Table 2:** Impact of metabolites on depression.

Metabolites	Impacts on depression	Mechanism	PMID
Tryptophan	A diet rich in tryptophan helps reduce depressive symptoms and improve an individual’s emotional state. A low tryptophan diet can lead to irritability and anxiety.	Tryptophan hydroxylase (TPH) enzyme plays an important role in various psychiatric disorders, including depression. The peripheral cells involved in the production of 5-HT showed TPH1 dysfunction, leading to insufficient levels of 5-HT in the brain, which in turn triggers a homeostatic response of TPH2. Excessive pro-inflammatory cytokines produced in depression over-activate the enzymes IDO and TDO, promoting the kynurenine pathway, thereby reducing the activation of the 5-HT pathway. Kynurenine can cross the blood–brain barrier and negatively impact brain health by inducing neuroinflammation and neurodegenerative changes.	25858202 28968985 16192985 25540092 25860609 25550456 18465467 22688187 27392632
SCFAs	Short-chain fatty acids shown to have antidepressant effects.	Butyrate administration can improve depression-related intestinal permeability and responsiveness of the HPA axis, resulting in an antidepressant effect. In humans and mice, an increase in dietary tryptophan induces the synthesis of SCFAs by the gut microbiota, resulting in increased production and release of 5-HT in ECs.	31646148 30066368 16945350 25860609 25550456 31216174 29902437
GABA	GABA has been shown to alleviate depressive-like behavior in mice.	JB-1 produce GABA, which crosses the intestinal barrier and is sensed by the vagus nerve, which activates the GABA signaling pathway, regulates the expression of GABAA and GABAB receptors and interacts with GABA. At the same time, the vagus nerve initiates neural activation, and PVN neurons initiate HPA axis activity to produce antidepressant effects.	21876150 27794467 30531975 22452873 32009871 29163522 26577887
Glycerophospholipid	An imbalance in hippocampal sphingolipid and glycerophospholipid metabolism associated with depressive-like behavior.	In a chronic unpredictable mild stress rat model of depression, a decrease in lipid metabolism-related enzymes associated with fatty acid synthesis and metabolism and glycerophospholipid metabolism was observed, indicating an imbalance in hippocampal sphingolipid and glycerophospholipid metabolism associated with depression-like behavior.	25754084 32376998 25803076 18755070 24590317
Choline	Choline deficiency can impair mental health, while excessive choline intake may lead to excessive synthesis of acetylcholine and result in depressive-like behavior.	Choline and its metabolites affect emotional behavior through DNA methylation. Supplementation with choline, betaine, and other methyl donors successfully reversed depression-like behavior. At the same time, cholinergic passage through the blood–brain barrier increases the concentration of acetylcholine in the brain, and abnormal choline metabolism may promote depression-like behavior.	28844887 25678811 26628207 10467961 14972364
Lactate	Lactate salts have a protective and reversing effect on depression.	Lactate can cross the blood–brain barrier and elevated levels of lactate have been observed in both germ-free rodents and MDD patients. Depression-like behavior was reversed by lactate administration.	2050746 30647450 28624318 28595107
Bile acids	Bile acid can cause severe depression, but some Bile acid, such as Ursodeoxycholic acid, show good antidepressant effect.	FXR is involved in bile acid synthesis, secretion, transport and regulation of CREB activity. By inhibiting CREB, bile acid can inhibit the transcription of BDNF, resulting in abnormal BDNF activity in patients with depression.	29163019 29677620 25870546 24629820
Vitamin (folate)	About one-third of depression patients exhibit folate deficiency, and administering folate in animal models of depression shows antidepressant effects.	Folate can synthesize BH4, which converts phenylalanine and tryptophan into the neurotransmitters dopamine, norepinephrine and 5-HT, thereby exerting an antidepressant effect. BDNF and GluR1 expression was also induced in the hippocampus and associated cortex.	18078962 20816716 10967371 2939126 1716662

## Microbiome-based clinical trials in depression

**Figure 3:**
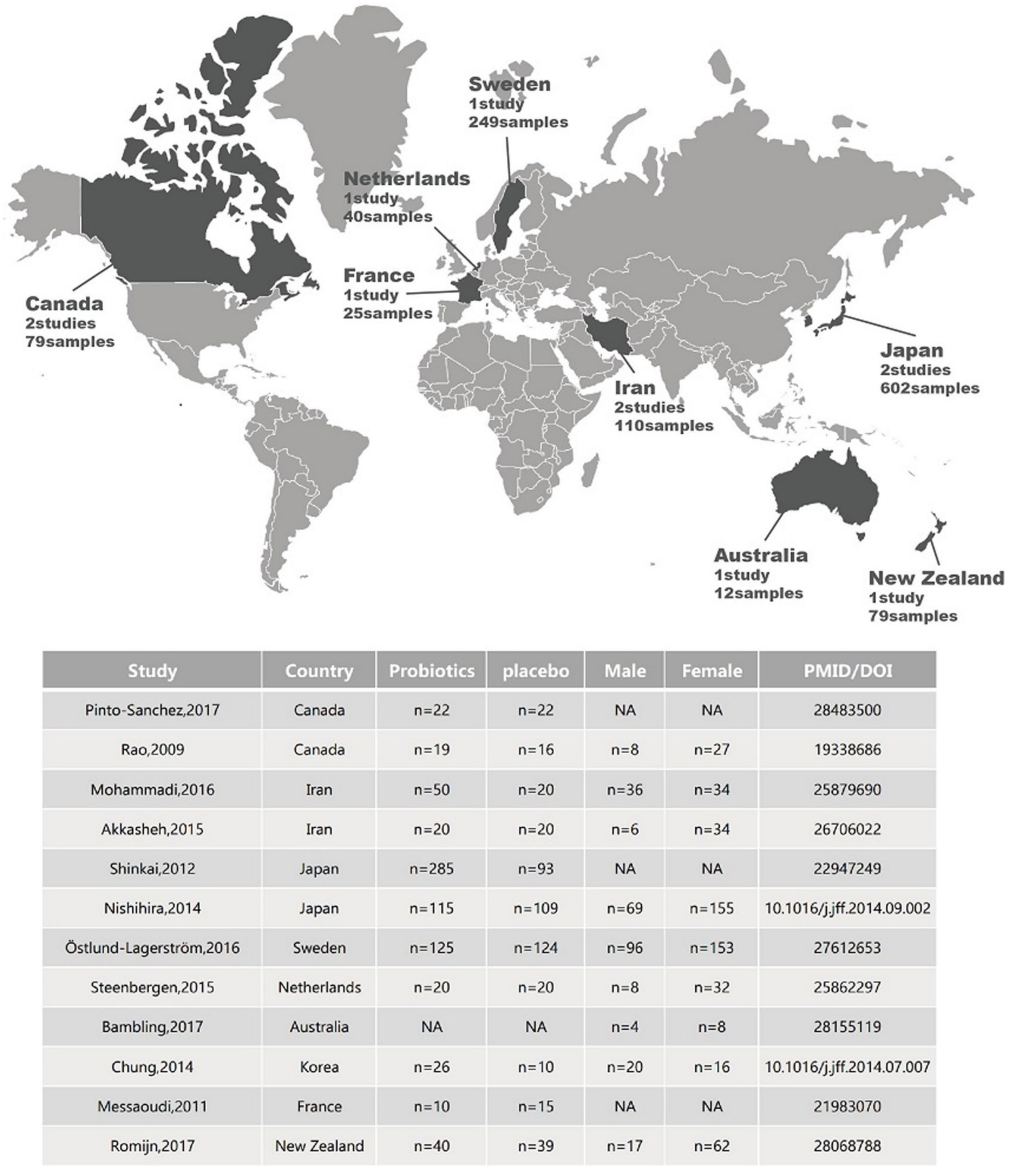
Geographical locations and sample metadata of different clinical trials related to depression.

**Figure 4:**
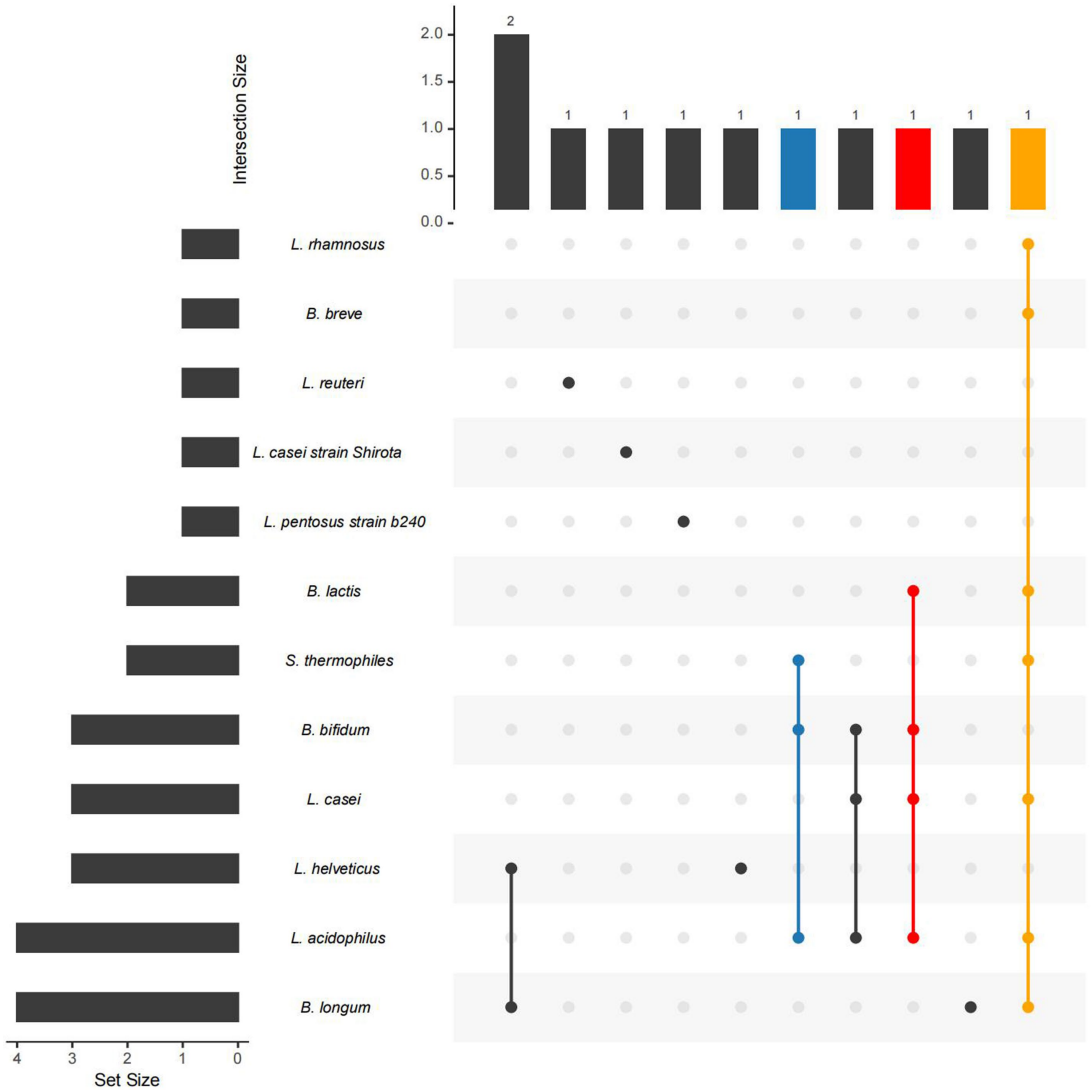
The occurrence of different probiotics across various studies. The left bar in the Upset Plot indicating the number of studies related to each microbe. Higher bars signify stronger evidence linking the microbe to depression. The upper bar displays the number of studies in which several connected microbes simultaneously used. A higher bar indicates that these microbes are found in a greater number of studies.

So far, probiotics have received significant attention as potential therapies for mood disorders and MDD (Figure 3; Table 3). The occurrence of different probiotics across various clinical trials was explored (Figure 4). *L. acidophilus* and *B. longum* are the most widely used probiotics in clinical trials. In healthy individuals, probiotic treatment with *L. helveticus* R0052 and *B. longum* R0175 for 30 days has been shown to reduce depression scores on the Hospital Anxiety and Depression (HAD) scale (Messaoudi et al., 2011). In patients with mild to moderate depression, a four-week treatment with a variety of probiotics (Bio-Kult Advanced^®^) significantly decreased PHQ9 scores (Baião et al., 2023). However, some studies have indicated that probiotic interventions may not effectively alleviate depressive symptoms. In individuals with moderate to severe depression, an eight-week administration of a probiotic mixture containing *L. helveticus* R0052 and *B. longum* R0175 as an adjunctive therapy did not improve depressive symptoms (Romijn et al., 2017). This study had a larger sample size and a longer treatment duration compared to previous successful trials of the same probiotic mixture in healthy subjects (Messaoudi et al., 2011). Probiotics are microbial preparations that, when ingested, can improve the balance of gut microbiota (Gibson and Roberfroid, 1995).

**Table 3:** Antidepressant effects of probiotics observed in clinical trials.

Probiotics	Administration form	Findings	Participant type	PMID/DOI
*B. longum* NCC3001 (BL)	Oral administration	BL reduces depression but not anxiety scores and increases quality of life in patients with IBS.	Adults with IBS and diarrhea or a mixed-stool pattern (based on Rome III criteria) and mild to moderate anxiety and/or depression.	28483500
*L. casei* strain Shirota (LcS)	Oral administration	Lactobacillus and Bifidobacteria significantly increased and symptoms of anxiety decreased in those taking LcS.	CFS patients	19338686
Probiotic yogurt contained *L. acidophilus* LA5 and *B. lactis* BB12. Conventional yogurt contained *S. thermophilus* and *L. bulgaricus.* Probiotic capsule contained *Lactobacillus casei*, *L. acidophilus*, *L. rhamnosus*, *L. bulgaricus*, *B. breve*, *B. longum*, and *S. thermophilus*	Oral administration of probiotic yogurt or probiotic capsule	After 6 weeks of intervention, a significant improvement of GHQ was observed in the probiotic yogurt and in the probiotic capsule group, as well as a significant improvement in DASS scores in the probiotic yogurt and the probiotic capsule group.	Petrochemical workers	25,879,690
*L. acidophilus, L. casei*, and *B. bifidum*	Oral administration of probiotic capsule with three viable and freeze-dried strains	8-week intervention decreased Beck Depression Inventory total scores, serum insulin concentration, homeostasis model assessment of insulin resistance, and serum hs-CRP concentration. Plasma total glutathione concentration was elevated.	Aged 20 to 55 with major depressive disorder (MDD).	26706022
*L. pentosus strain* b240	Oral administration of heat-killed probiotics	Oral probiotics significantly reduced the incidence of the common cold in elderly adults.	Elderly adults aged 65 and above	22947249
*L. gasseri* SBT2055 and *B. longum* SBT2928	Oral administration of yogurt containing two different probiotics	Probiotics enhanced immunity and alleviated stress.	Healthy adults aged 32 to 76.	10.1016/j.jff.2014.09.002
*L. reuteri* DSM17938	Oral administration	No persistent significant effects were observed on the primary or secondary outcome measures of the study.	General elderly	27612653
*Bifidobacterium (B.) bifidum* W23*, B. lactis* W52*, Lactobacillus (L.) acidophilus* W37*, L. brevis* W63*, L. casei* W56*, L. salivarius* W24, and *Lactococcus lactis* (W19 and W58)	Oral administration of freeze-dried powder of the probiotic mixture	Consumption of multiple probiotics for 4 weeks significantly reduced overall cognitive responses to depression, especially aggression and rumination.	No psychiatric or neurological disorders, no personal or family history of depression or migraines.	25862297
*L. acidophilus, B. bifidum, Streptoccocus (S.) thermophiles*	Probiotics/magnesium orotate formulation adjuvant administered with SSRIs	At the end of an 8-week intervention mean changes for depression scores and quality of life in the group was clinically significantly improved. The participants who responded to treatment reported a subjective increase in well-being and improved energy levels.	Meets criteria for resistant depression, is currently taking antidepressants, and has a history of multiple depressive episodes that have poor response to treatment.	28155119
*L. helveticus* IDCC3801	Oral administration of fermentation of milk using probiotic Lactobacillus suis IDCC3801 (LHFM)	Cognitive tests improved after 12 weeks of LHFM administration.	Healthy elderly people aged 60 to 75	10.1016/j.jff.2014.07.007
*L. helveticus* R0052 and *B. longum* R0175	Oral administration	*Lactobacillus helveticus* R0052 and *Bifidobacterium longum* R0175 (PF) taken in combination for 30 days decreased the global scores of hospital anxiety and depression scale (HADs), and the global severity index of the Hopkins symptoms checklist (HSCL90).	General population	21983070
*L. helveticus, B. longum*	Oral administration	No significant evidence was found effectively treating low mood or modcrating the levels of inflammatory and other biomarker.	Not currently taking psychotropic drugs and scoring at least moderate on a self-reported emotional measurement.	28068788

Details of the samples used in these studies can be found in Figure 2.

Prebiotics are compounds that are not broken down, absorbed, or utilized by the human body but promote the growth of gut microbiota, ultimately benefiting the host (Valcheva and Dieleman, 2016). Compounds evaluated in prebiotic trials include Bimuno^®^-galactooligosaccharide (B-GOS), fructooligosaccharide (FOS), GOS, and short-chain FOS (scFOS). None of the five depression prebiotic trials (Smith, 2005; Silk et al., 2009; Smith et al., 2015; Azpiroz et al., 2017; Kazemi et al., 2019) demonstrated a significant impact on depressive symptoms. Two studies examining the benefits of prebiotic treatment also did not observe a decrease in depression symptom scores during the eight-week follow-up period (Heidarzadeh-Rad et al., 2020; Vaghef-Mehrabany et al., 2021). When studied as standalone therapies, prebiotics yielded non-statistically significant results (Akkasheh et al., 2016; Kazemi et al., 2019; Rudzki et al., 2019). However, no studies have indicated any negative effects of probiotic/prebiotic interventions on depressive symptoms.

The combination of probiotics and prebiotics is known as synbiotics (Sorbara and Pamer, 2022). In a single synbiotic study by Ghorbani et al., a reduction in depressive symptoms was observed after 8 weeks of synbiotic treatment (Ghorbani et al., 2018). In a recent systematic review and meta-analysis by Hofmeister et al., evidence from 50 randomized controlled trials (RCTs) evaluating probiotics, prebiotics, synbiotics, postbiotics, or fecal microbiota transplantation interventions in adult populations was synthesized. Improvement in depressive symptoms was reported based on the Beck Depression Inventory (BDI) and the depression subscale of the Hospital Anxiety and Depression Scale (Hofmeister et al., 2021).

Current evidence suggests that prebiotics as standalone therapies are unlikely to be effective for depression (Akkasheh et al., 2016; Ghorbani et al., 2018; Majeed et al., 2018; Miyaoka et al., 2018; Kazemi et al., 2019). While probiotics and synbiotics appear to be effective in alleviating depressive symptoms, the evidence supporting this observation is mixed. One possible explanation for these mixed findings is that individuals with mild depression may derive more benefits from probiotic and synbiotic treatments compared to those with chronic treatment-resistant depression (Wallace and Milev, 2021). Further studies focusing on different levels of depression severity would help clarify the benefits of these treatments. Additionally, the use of prebiotic and synbiotic therapies for depression is largely understudied, and the evidence is not as specific, necessitating multiple studies analyzing each compound.

## Prospects and summary

The gut microbiota plays a crucial role in regulating human health. Extensive research has shown that the gut microbiota can influence the occurrence and development of depression through the MGB axis, involving neural, immune, and especially metabolic pathways. In reported studies, the gut microbiota has been found to play a significant role in the onset and progression of mental disorders, including depression. For example, when the microbiota from individuals with depression is transplanted into healthy animals, it can induce depression-like behaviors. At the same time, the microbiota can also improve depression-like behaviors. In animal studies, the administration of probiotics has been shown to significantly improve depression-like behaviors in both rats and mice.

Diagnosis and treatment of depression based on the gut microbiota is considered a future research direction. In human studies, administration of probiotics has shown some degree of effectiveness in alleviating depression symptoms. However, prebiotics as a standalone therapy for depression are unlikely to be effective, and the combination of probiotics and prebiotics has not demonstrated significant symptom relief. Yet, there is still limited research on the use of prebiotics and synbiotics in the treatment of depression, and exploring these therapies may uncover beneficial effects.

The CRISPR/Cas9 system is a potent genome editing tool widely utilized in basic, preclinical, and clinical studies as extensively reviewed for genetic disorders (Zhang et al., 2023). While limited studies have employed CRISPR/Cas9 in depression-associated research, there are already CRISPR/Cas-based genome editing tools for *Bacteroides* (Zheng et al., 2022). These tools significantly aid mechanistic studies of gut commensals and the development of engineered live biotherapeutics.

Constrained-based modeling (Heinken and Thiele, 2015) allows for the versatility needed to simulate bacterial communities under various conditions that cannot be replicated *in vivo*, such as *C. difficile* infection. Thus, by employing computational modeling of microbial metabolism using software like MICOM (Diener et al., 2020), a framework is provided to infer the growth rates of selected bacteria and the metabolic interactions within the gut microbiota. Additionally, it offers a high-throughput platform for generating mechanistic hypotheses and testing them in clinical analyses. We believe that the most valuable application of metabolic models in bacterial communities is to provide detailed functional metabolic inferences as a means of testing new hypotheses, thereby laying the groundwork for the development of more accurate models. While these computational approaches have been applied to the study of the gut microbiota, their use in exploring the metabolic consequences of depression is groundbreaking. The outcomes of this approach include the development of more precise models by incorporating information about the ecological relationships between the gut microbiota and its host. From a metabolic perspective, integrating individual differences in genetics and gut microbiota holds promise for personalized recommendations of effective therapies for depression.

## Author contributions

MW: Writing – original draft. ZS: Writing – original draft. SL: Writing – original draft, Data curation, Validation, Visualization. FT: Writing – review & editing, Methodology, Supervision, Validation, Investigation. LD: Funding acquisition, Writing – review & editing. FY: Conceptualization, Funding acquisition, Project administration, Writing – review & editing, Writing – original draft.
